# Risk factors for 3-year mortality in selected patients with Parkinson’s disease from a Romanian cohort

**DOI:** 10.25122/jml-2024-0332

**Published:** 2024-07

**Authors:** Diana Sipos-Lascu, Ștefan Cristian Vesa, Ionel-Lucian Stan, Nicu-Catalin Draghici, Lacramioara Perju-Dumbravă

**Affiliations:** 1 Department of Neurosciences, Iuliu Hațieganu University of Medicine and Pharmacy, Cluj-Napoca, Romania; 2 Department of Pharmacology, Toxicology and Clinical Pharmacology, Iuliu Hatieganu University of Medicine and Pharmacy, Cluj-Napoca, Romania; 3 Department of Medical Specialties/Epidemiology, Iuliu Hațieganu University of Medicine and Pharmacy, Cluj-Napoca, Romania; 4 Emergency Room, Cluj-Napoca Emergency Clinical County Hospital, Cluj-Napoca, Romania; 5 IMOGEN Institute, Emergency Clinical County Hospital Cluj, Cluj-Napoca, Romania; 6 Neurology Clinic, Cluj-Napoca Emergency Clinical County Hospital, Cluj-Napoca, Romania

**Keywords:** Parkinson’s disease, mortality, levodopa dosage, anhedonia, predictive factors

## Abstract

This study aimed to identify and analyze factors associated with a higher risk of 3-year mortality in patients with Parkinson’s disease (PD) within a Romanian cohort, focusing on individuals with more advanced disease stages as indicated by the Hoehn and Yahr scale. We conducted a cross-sectional observational study on 42 patients with PD treated at the Neurology Clinic I, Cluj-Napoca County Emergency Clinical Hospital, between October 2019 and January 2021. All participants were at stages 2.5 or 3 on the Hoehn and Yahr scale at baseline. Various clinical, neuropsychological, and neurophysiological assessments were performed, including evaluations for motor and non-motor symptoms such as anhedonia (via the Snaith-Hamilton Pleasure Scale - SHAPS) and cognitive impairment. The use of antiparkinsonian medications and antidepressants was also recorded. Factors associated with higher mortality risk included a higher anhedonia score (SHAPS > 34; *P* = 0.03), higher levodopa doses (cutoff = 937.5 mg; *P* = 0.001), and the administration of mirtazapine (*P* = 0.04). These findings indicate that non-motor symptoms like anhedonia, along with higher medication doses and specific treatments, play a significant role in influencing mortality risk in advanced PD. This study highlights the multifaceted nature of mortality risk in patients with PD, particularly emphasizing the role of non-motor symptoms and pharmacological treatment. Tailored therapeutic strategies, including closer monitoring of anhedonia and careful management of medication dosages, may be essential in reducing mortality and improving patient outcomes in advanced stages of PD.

## INTRODUCTION

Parkinson’s disease (PD) is a chronic, progressive neurodegenerative disorder that affects millions of individuals worldwide [1]. Although primarily characterized by its impact on motor function, PD encompasses a wide range of motor and non-motor symptoms that significantly affect patient morbidity and mortality [2]. With advancements in medical care, patients are living longer with PD, but the progression of the disease and the development of associated complications have led to an increased focus on mortality and its risk factors [3].

Numerous studies have aimed to identify the factors contributing to increased mortality in PD patients, recognizing that these insights are vital for improving patient management and outcomes. Among the most notable risk factors are older age at disease onset, motor impairments, and the presence of various non-motor symptoms such as psycho-affective disorders, cognitive decline, psychosis, and autonomic dysfunction. Additionally, complications such as respiratory issues, cardiovascular disease, and malnutrition play a critical role in patient survival [4,5].

Some medications have been mentioned in the literature as being associated with the risk of mortality in these patients, noting that some of them are used in high doses predominantly in the advanced stages of the disease when the association of complications itself increases this risk [6]. Understanding these risk factors allows for a more holistic approach to PD treatment, addressing both the direct impact of the disease and the secondary conditions that contribute to increased mortality.

This article aims to highlight the factors associated with a higher risk of 3-year mortality, identified in a Romanian cohort, based on a baseline consisting of patients who, at the time of examination, had progressed to a more advanced disease stage on the Hoehn and Yahr scale, specifically stages 2.5 or 3.

## MATERIAL AND METHODS

The present study was a cross-sectional, observational study and included 42 patients with Parkinson’s disease who presented at the Neurology Clinic I, Cluj-Napoca County Emergency Clinical Hospital, between October 1, 2019, and January 15, 2021.

The inclusion criteria were as follows: patients with Parkinson’s disease who had progressed to a Hoehn and Yahr score of 2.5 or 3 at the time of evaluation, with or without antidepressant therapy, and who provided signed informed consent documents for voluntary participation in the study. Exclusion criteria included patients with Hoehn and Yahr stage 4 or 5, those in a state of grief, and individuals who either did not sign the informed consent form for participation in the study and/or the agreement regarding the processing of personal data for research purposes. The study was approved by the ethics committee of the Iuliu Hațieganu University of Medicine and Pharmacy in Cluj-Napoca, Romania (121/2021). After collecting anamnesis data, neurological evaluations, and calculating the Unified Parkinson’s Disease Rating Scale (UPDRS) score, patients were classified by disease severity according to the Hoehn and Yahr scale [7,8].

All patients underwent psychological evaluation, including cognitive assessment, using the Mini-Mental State Examination (MMSE) scale [9]. Psychiatric examinations were performed on patients whose psychological evaluations revealed depressive elements, increased emotional reactivity, moderate/severe anxiety (according to the Leahy Anxiety Scale), or moderate/severe cognitive impairments (according to MMSE) [9,10]. Patients were assessed using apathy scales: Lille Apathy Rating Scale (LARS), Apathy Evaluation Scale (AES), Dimensional Apathy Scale (DAS), and UPDRS part I item 4, as well as the Snaith–Hamilton Pleasure Scale (SHAPS) for anhedonia [7,11-14]. Cutoff values determined in validation studies for these scales were used [7, 11-14].

Patients who met the inclusion criteria underwent P100 wave measurement using a standardized device for evoked potential responses (Keypoint 4, Medtronic, Denmark; software: Keypoint v. 5.11- Alpine BioMed) via the 'Reversal Pattern’ technique. The reversal rate was set at 2 Hz. Each subject was tested on both eyes individually, with the other eye covered during the test. Latencies of N75, P100, and N135 waves and the amplitude of the P100 wave were recorded. All our scores and neurophysiological tests were performed while patients with motor fluctuations were in the 'on’ phase. Three years after the patients had progressed to Hoehn and Yahr stages 2.5 or 3, their survival status was verified using the National Health Insurance House of Romania’s website based on their personal identification number (http://cas.cnas.ro/page/verificare-asigurat.html).

Data analysis was performed using MedCalc Statistical Software version 23.0.2 (MedCalc Software Ltd, Ostend, Belgium; https://www.medcalc.org; 2024). Continuous variables were expressed as medians and interquartile ranges due to their non-normal distribution, which was confirmed using the Shapiro-Wilk test. Nominal variables were presented as frequencies and percentages. Comparisons between deceased and surviving groups were made using the Mann-Whitney U test for continuous variables and the Chi-square or Fisher’s exact test for categorical variables, as appropriate. A cutoff value for levodopa dosage and the anhedonia score was determined using the Receiver Operating Characteristic (ROC) curve analysis, and the Area Under the Curve (AUC) was calculated along with sensitivity and specificity. Multivariate logistic regression analysis was employed to identify independent predictors of 3-year mortality. A *P* value of less than 0.05 was considered statistically significant.

## RESULTS

There were ten patients who died during the follow-up period. Comparisons between the deceased and surviving patients are presented in Table 1. Deceased patients were more likely to follow treatment with mirtazapine (*P* = 0.008), to have a higher dose of Levodopa (*P* = 0.002), or to have a high anhedonia SHAPS score (*P* = 0.01).

**Table 1:** Demographic, clinical, and treatment characteristics of deceased vs. surviving patients

Variables		Survivors (*n* = 32)	Deceased (*n* = 10)	*P*
Age		65.5 (61;71)	72.5 (66.7;76)	0.06
Age of onset		56.5 (51.2;60)	61 (56.7;66.2)	0.1
Environment	Rural	3 (9.4%)	1 (10%)	1
	Urban	29 (90.6%)	9 (90%)	
Gender	Male	16 (50%)	8 (80%)	0.1
	Female	16 (50%)	2 (20%)	
Smoking	No	23 (71.9%)	8 (80%)	1
	Yes	9 (28.1%)	2 (20%)	
Coffee consumption	No	8 (25%)	5 (50%)	0.2
	Yes	24 (74%)	5 (50%)	
Depression	No	32 (100%)	4 (40%)	0.1
	Yes	10 (31.1%)	6 (60%)	
Rotigotine	No	24 (75%)	9 (90%)	0.4
	Yes	8 (25%)	1 (10%)	
Rasagiline	No	18 (56.3%)	7 (70%)	0.4
	Yes	24 (43.8%)	3 (30%)	
Amantadine	No	27 (84.4%)	9 (90%)	1
	Yes	5 (15.6%)	1 (10%)	
Mirtazapine	No	31 (96.9%)	6 (60%)	0.008
	Yes	1 (3.1%)	4 (40%)	
Arterial hypertension	No	13 (40.6%)	5 (50%)	0.7
	Yes	19 (59.4%)	5 (50%)	
Diabetes mellitus	No	29 (90.6%)	8 (80%)	0.3
	Yes	3 (9.4%)	2 (20%)	
Levodopa dose		750 (375; 937)	1000 (750; 1500)	0.002
UPDRS scale		38.5 (25.5;44)	47 (35.5;57)	0.1
MMSE		28.5 (27;30)	27 (25.7;29)	0.09
Apathy score DAS		10.5 (6.25; 17)	9 (6;20.5)	0.9
Anhedonia score SHAPS		23 (16; 32)	49 (19.5;51.5)	0.01
Latency P100 for the right eye		110 (99; 117.5)	115.7 (98.5;118.2)	0.7
Latency P100 for the left eye		109.5 (100;117.5)	109 (99.7;117.1)	0.9
Difference in latency between eyes		2 (1;5.3)	2.1 (0.5;4.5)	0.5
Predominant side	Symmetric	4 (12.5%)	4 (40%)	0.1
	Right	15 (46.9%)	4 (40%)	
	Left	13 (40.6%)	2 (20%)	

UPDRS, Unified Parkinson’s Disease Rating Scale; MMSE, Mini-Mental State Examination; LARS, Lille apathy rating scale; DAS, Dimensional Apathy Scale; SHAPS, Snaith-Hamilton Pleasure Scale.

A cutoff value of 937.5 mg for levodopa dose was calculated as a predictor of 3-year mortality (AUC = 0.781 (95% CI, 0.627–0.894); Se = 60 (95% CI, 26.2–87.2), Sp = 71.8 (95%CI, 53.3–86.3); *P* = 0.001). For the SHAPS anhedonia score, a cutoff value of 34 was calculated for 3-year mortality (AUC = 0.767 (95% CI, 0.611–0.883); Se = 70 (95% CI, 34.8–93.3), Sp = 84.3 (95%CI, 67.2–94.7); *P* < 0.001).

Multivariate logistic regression was used to determine the variables independently associated with mortality. A high SHAPS score (*P* = 0.03) and treatment with mirtazapine (*P* = 0.04) were independent predictors of long-term mortality in patients with PD (Table 2).

**Table 2:** Multivariate logistic regression for 3-year mortality

Variables	B	*P*	OR	95% C.I. for OR	
				Min	Max
Anhedonia score SHAPS>34	2.108	0.03	8.232	1.107	61.226
Levodopa dose>937.5 mg	1.088	0.2	2.969	0.405	21.765
Mirtazapine	2.759	0.04	15.778	1.043	238.581
Constant	0.121	0.8	1.128		

SHAPS, Snaith-Hamilton Pleasure Scale

## DISCUSSION

Mortality risk factors for patients with Parkinson’s disease have been the subject of various studies, as PD is a complex neurodegenerative disorder with numerous factors influencing both disease progression and overall survival. Commonly identified high-risk factors for mortality in Parkinson’s disease (PD) include older age at onset or diagnosis, longer disease duration (due to complications such as falls, infections, and worsening motor symptoms), motor impairments (which increase the likelihood of falls and immobility), freezing of gait and frequent falls (leading to injuries, fractures, and head trauma), non-motor symptoms like cognitive impairment, psychosis, depression, and anxiety, autonomic dysfunctions (such as dysphagia, orthostatic hypotension, urinary incontinence, and constipation), immobilization and frailty, respiratory complications (such as aspiration pneumonia and sleep apnea), cardiovascular issues, poor response to levodopa, comorbidities like diabetes, heart disease, and hypertension, malnutrition and cachexia (muscle wasting), male gender (slightly higher risk), low body mass index (BMI), and the presence of certain genetic mutations (like LRRK2, PINK1, and GBA genes). These factors can interact in complex ways, and understanding the relative impact of each can help tailor treatment and care strategies for individuals with PD to potentially reduce mortality risk [4,15,16].

Anhedonia, the reduced ability to experience pleasure or interest in activities the patient previously enjoyed, is a common non-motor symptom of PD. It is often associated with depression and can significantly impact the quality of life [17,18]. While anhedonia itself was not directly linked to increased mortality in patients with PD in previous studies, it was proved to contribute to mortality indirectly due to the possible association with depression (patients with anhedonia and depression being less likely to adhere to treatment, engage in physical activity, or maintain good nutrition), reduced engagement in activities (which can contribute to muscle wasting, immobility, and increased risk of falls), malnutrition and weight loss (reduced interest in food, increasing susceptibility to infections and frailty) and cognitive decline (or dementia)[17,18]. In our study, the presence of anhedonia, evaluated using the SHAPS scale (with a point-based scoring system ranging from 14 to 56, with higher scores indicating more severe anhedonia), was correlated with an increased risk of mortality (*P* = 0.03) at a cutoff value of 34. We mention that this cutoff value is similar to the one frequently used in the literature to indicate significant anhedonia (≥ 36) [14,17].

Certain medications prescribed to patients with PD, particularly antipsychotic drugs, have also been shown to increase mortality. A study found that patients using antipsychotic medications had a 2.35 times higher mortality rate than those not taking them. First-generation antipsychotics, like haloperidol, were associated with the highest risk, increasing mortality by up to five times compared to no treatment. Even atypical antipsychotics like risperidone and quetiapine were linked to elevated risks, though slightly lower than the older medications [19,20]. Regarding levodopa, higher doses, particularly those exceeding a levodopa equivalent daily dose of 600 mg, have been associated with an increased risk of mortality in patients with PD [21]. This higher mortality risk may be linked to disease severity, as patients requiring higher doses often exhibit more advanced symptoms, such as gait disturbances and postural instability. However, it remains unclear whether the increased mortality is directly caused by the medication itself or by the underlying disease progression that necessitates higher doses of levodopa [22]. This study evaluated whether levodopa dosage correlates with an increased mortality risk in the enrolled patients. We found that a daily cutoff dose value of 937.5 mg was significantly associated with higher mortality (*P* = 0.001), consistent with results from other populations in the literature. The plausible explanation is that greater disease severity, often associated with complications, necessitates higher doses of levodopa.

A statistically significant association between mortality and the administration of mirtazapine was observed in our cohort (*P* = 0.04). Mirtazapine is an antidepressant drug that belongs to the class of noradrenergic and specific serotonergic antidepressants, often prescribed for treating depression, anxiety, and sleep disturbances in various populations, including patients with PD. In these individuals, mirtazapine is sometimes used to address common non-motor symptoms like depression and anxiety, also prescribed for sleep disorders, which many patients with PD experience [23]. However, its use must be carefully monitored due to the complexity of PD and the potential interactions with other medications used to manage PD motor symptoms, such as levodopa. Although mirtazapine is not specifically shown to increase mortality in patients with PD, other studies have raised concerns about increased all-cause mortality associated with its use in certain populations. This is especially important for patients with comorbidities like cardiovascular disease [19].

## CONCLUSION

The findings from our study underline the critical impact of non-motor symptoms, particularly anhedonia, and the effects of pharmacological treatments on the mortality of patients with PD. Anhedonia, as assessed by the SHAPS scale, emerged as a significant predictor of 3-year mortality, reinforcing the need to consider psychological health alongside motor symptoms in the comprehensive management of PD. Additionally, the relationship between higher doses of levodopa and mortality highlights the necessity for careful dose management, particularly in patients with advanced disease stages. The observed association between mirtazapine administration and increased mortality warrants further investigation, particularly in light of its frequent use for non-motor symptoms in PD.

These results suggest that addressing non-motor symptoms and optimizing pharmacological treatments are crucial for improving survival in patients with PD. Personalized care strategies that incorporate these elements may help mitigate the risk of mortality and enhance the quality of life for individuals living with PD. Further research is necessary to elucidate the underlying mechanisms driving these associations and to refine treatment protocols for patients with PD at high risk of mortality.
